# Behaviour of Human Induced Pluripotent Stem Cell-Derived Neural Progenitors on Collagen Scaffolds Varied in Freezing Temperature and Laminin Concentration

**Published:** 2014-02-03

**Authors:** Fahimeh Khayyatan, Shiva Nemati, Sahar Kiani, Shahriar Hojjati Emami, Hossein Baharvand

**Affiliations:** 1 Department of Stem Cells and Developmental Biology at Cell Science Research Center, Royan Institute for Stem Cell Biology and Technology, ACECR, Tehran, Iran; 2 Department of Biomedical Engineering, Amirkabir University of Technology, Tehran, Iran; 3 Department of Developmental Biology, University of Science and Culture, ACECR, Tehran, Iran

**Keywords:** Collagen, Laminin, Neural Progenitors, Tissue Engineering

## Abstract

**Objective:**

Biomaterial technology, when combined with emerging human induced pluripotent stem cell (hiPSC) technology, provides a promising strategy for patient-specific tissue engineering. In this study, we have evaluated the physical effects of collagen scaffolds fabricated at various freezing temperatures on the behavior of hiPSC-derived neural progenitors (hiPSC-NPs). In addition, the coating of scaffolds using different concentrations of laminin was examined on the cells.

**Materials and Methods:**

Initially, in this experimental study, the collagen scaffolds fabricated from different collagen concentrations and freezing temperatures were characterized by determining the pore size, porosity, swelling ratio, and mechanical properties. Effects of cross-linking on free amine groups, volume shrinkage and mass retention was also assessed. Then, hiPSC-NPs were seeded onto the most stable three-dimensional collagen scaffolds and we evaluated the effect of pore structure. Additionally, the different concentrations of laminin coating of the scaffolds on hiPSC-NPs behavior were assessed.

**Results:**

Scanning electron micrographs of the scaffolds showed a pore diameter in the range of 23-232 μm for the scaffolds prepared with different fabrication parameters. Also porosity of all scaffolds was >98% with more than 94% swelling ratio. hiPSC-NPs were subsequently seeded onto the scaffolds that were made by different freezing temperatures in order to assess for physical effects of the scaffolds. We observed similar proliferation, but more cell infiltration in scaffolds prepared at lower freezing temperatures. The laminin coating of the scaffolds improved NPs proliferation and infiltration in a dose-dependent manner. Immunofluorescence staining and scanning electron microscopy confirmed the compatibility of undifferentiated and differentiated hiPSC-NPs on these scaffolds.

**Conclusion:**

The results have suggested that the pore structure and laminin coating of collagen scaffolds significantly impact cell behavior. These biocompatible three-dimensional laminin-coated collagen scaffolds are good candidates for future hiPSC-NPs biomedical nerve tissue engineering applications.

## Introduction

The properties of self-renewal and pluripotency of human embryonic stem cells (hESCs) and human induced pluripotent stem cells (hiPSCs) have paved the way for the generation of neural progenitors (NPs) (1, 2). *In vitro* differentiation of hESCs to NPs and neural cells serves as a model for the study of early human neuronal development and potentially offers an unlimited cell source for drug screening and cell-based therapies. The combination of NPs with tissue engineering provides a promising future for novel cell transplantationbased therapies (3). We have previously shown that hESC-derived NPs (hESC-NPs) in threedimensional collagen display neuronal differentiation with typical synapses (4). We found that hESC-NPs cultured in collagen caused improvement in an injured spinal cord model in rats (5).

Novel neural tissue engineering needs to address several issues before in vivo engraftment of NPs to ensure their successful incorporation, survival, and functional integration into diseased or injured regions of the central nervous system (6). One critical element is the regulation of interactions between scaffolds and cells with the intent to provide a microenvironment that mimics numerous characteristics of natural extracellular matrices (ECMs). To achieve this goal, physical (7), chemical (8) and mechanical (9) properties of scaffolds have to be taken into consideration.

Physical properties of tissue-engineered scaffolds such as pore size, porosity, pore shape and orientation have been shown to influence cellular behavior (7). The average pore size should be optimal for cell migration and provide a suitable surface area for cell attachment, which varies with different cell types (10). High porosity and interconnectivity is also important for cells and metabolite transport, however it may alter mechanical properties. Pore shape is another physical cue that can affect cell morphology and modulate cellular responses *in vitro*. Cells align with the axis in the oriented pores, which is crucial for neural tissue engineering to direct neurites (11).

Biochemical aspects of the ECM are another essential prerequisite for neural tissue engineering that must be taken into consideration. Collagen and laminin are major components of the neural ECM that have a high impact in enhancing neural cell activity (12). Collagen is a naturally derived polymer that has the potential advantage of specific cell interactions in addition to a hydrophilic nature, yet it possesses poor mechanical properties (13). Collagen is commonly used as scaffolding material in tissue engineering because it has numerous advantageous properties, which include low antigenicity and high cell growth promotion. On the other hand, laminin has a significant role in neurogenesis and neural development, thus biomaterial engineers try to use this natural biomaterial for neural tissue engineering in different forms, such as threedimensional scaffolds (14), nanofiber meshes (15), and as coating material (16). Although physical or biochemical aspects of two-dimensional substrates on cell migration have been widely investigated, the effects of these aspects on three-dimensional scaffolds have been less studied.

This study aims to investigate the effects of the physical structure of collagen scaffolds at various freezing temperatures. We have also evaluated the biochemical coating of these scaffolds by using different concentrations of laminin with the intent to examine its effect on the proliferation and infiltration of hiPSC-NPs as candidates for novel patient-specific cell therapies of neural defects. The combination of these two major ECM components (collagen and laminin) in the design of scaffolds could be an appropriate strategy to better mimic neural cells microenvironment.

## Materials and Methods

### Fabrication of a collagen sponge

Collagen type I was derived from the tendon of a rat’s tail (17). We prepared 0.3, 0.5, and 1% (w/v) suspensions by the incubation of insoluble type I collagen in 0.5 M acetic acid (pH=2.5) overnight at 4˚C and homogenization, after the addition of ice-cold distilled water. The suspension was filtered through a 100 μm nylon filter (BD Falcon™ Cell Strainers, BD Biosciences, USA) and de-aerated under vacuum. Next, 0.75 ml of the collagen solution was poured into a polystyrene mold (24-well TPP culture plate, Switzerland) and frozen at temperatures of -20, -80, and -196˚C in liquid nitrogen. The cooling rate was not controlled. After freezing, samples were lyophilized for 24 hours. For cross-linking of the collagen sponges, small volumes of chilled acetone were added first, then allowed to react with a 0.6% wt glutaraldehyde (GA, 25%, EM grade, TAAB, UK) solution for 24 hours at 4˚C with vigorous stirring. After 24 hours of the cross-linking reaction, cross-linked collagen sponges were removed from the 4˚C environment (characterized by a yellow solution) and added to 0.1 M glycine (G8790, Sigma- Aldrich) solution for 1 hour at room temperature to stop the reaction. Sponges were then rinsed three times with double distilled water, frozen, and subsequently freeze-dried for 48 hours.

The effect of cross-linking on the samples was evaluated by using the following equation: Volume shrinkage (%)=[1- (V/V0)]×100 Where V is the volume of the scaffold after crosslinking and V0 is the original volume.

Mass retention (%)=(massafter cross-linkingmassbefore cross-linking)×100

### Structure and morphology

The structure and morphology of the platecontacting surface of samples were studied using a scanning electron microscope (SEM, VEGA\ TESCAN, Czech Republic) at an operating voltage of 15 kV. Freeze-dried samples were mounted on stubs and sputtered with an ultrathin layer of gold in an ion sputter (EM/TECH, K 350, UK) at 20 mA for 4 minutes. To determine average pore sizes, we measured the dimensions of at least 50 randomly chosen pores from the samples by image analyzer program measurement (Image J). The porosity of the scaffolds was calculated by determining the volume (V) and the mass (m) of the scaffolds. Porosity was defined as:

p (%)=[1–(ddp)]×100

Where_d_ was the density of the scaffold and dp was the density of the polymer (1.32 g/cm3) (18).

### Structure and morphology

Collagen scaffolds were separately immersed in double distilled water at room temperature for 24 hours. After excess water was removed, we determined the wet weight of the scaffold (Ws). The water uptake of the scaffold was calculated as follows:

Swelling ratio (%)=[(Ws-Wd)Ws]×100

Where Wd was the weight of the dry scaffold.

### Determination of primary amine group content

The concentrations of free primary amine groups present in cross-linked and native collagen were determined using 2, 4, 6-trinitrobenzenesulfonic acid (TNBS, 28997, Pierce, USA) as previously described (19). Briefly, samples were incubated in 4% (w/v) NaHCO3 followed by addition of 0.5% (w/v) TNBS solution, then incubation at 40˚C for 2 hours. After the addition of 2 ml HCl (6 M), the reaction mixture was diluted with demineralized MilliQ water, cooled to room temperature, and its absorbance was measured at 420 nm. HCl was added before TNBS as a blank and the calibration curve was obtained with glycine.

### Mechanical properties

Stress-strain properties of flat freeze-dried scaffolds were determined using a computer-controlled Instron mechanical tensile tester (5566 series, UTM, Instron Corporation, UK) that had a grip-to-grip separation of 15 mm and was operated at a crosshead speed of 5 mm/minute at room temperature. A load cell of 50 N was used. We used a predesigned blade knife to cut bone-shaped flat specimens from the scaffolds. The thickness of the samples in the test area was measured at three different points using a thickness gauge (SDL International Ltd., UK). Young’s modulus, maximum load (UTS), and strain at failure were calculated from the stress-strain curves.

### Human-induced pluripotent stem cell-derived neural progenitors culture (hiPSC-NPs)

NPs were generated from Royan hiPSC4 (20) as previously described (2). NPs were cultured in a neural expansion medium that contained DMEM-F12 medium supplemented with 5% knockout serum replacement (KOSR), 1% non-essential amino acid, 2 mM L-glutamine, 2% N2 (all from Invitrogen), 0.1 mM β-mercaptoethanol, 20 ng/ml basic fibroblast growth factor (bFGF, Royan Institute), 20 ng/ml additional epidermal growth factor (EGF, Sigma-Aldrich) and 0.2 mM ascorbic acid (Sigma-Aldrich). The media was changed every other day. Cells were passaged at high cell density, typically three days after replating. Passages were treated with 0.5% trypsin/0.53 mM EDTA (Invitrogen) and split at 1:2-1:3 ratios. The cells were re-plated on 1 μg/ml laminin and 15 μg/ml poly- L-ornithine (both from Sigma-Aldrich)-coated tissue culture dishes in the same medium. Spontaneous differentiation was performed in a differentiation medium in the absence of growth factors, but included neurobasal medium (Invitrogen) and DMEM-F12 (1:1), B27 (1%), KOSR (5%), and N2 supplement (1%). Half of the medium was renewed every five days.

### Seeding of hiPSC-NPs

We chose scaffolds prepared with a 1% collagen solution and GA cross-linking for cell seeding because of their high stability. The matrices were sterilized with 70% (v/v) ethanol, and then rinsed extensively three times with sterile phosphate buffered saline (PBS). For laminin coating, the scaffolds were immersed in 1 ml each of 0, 1, 10, and 20 μg/ml laminin in PBS, overnight at 4˚C. These laminin concentrations are widely used for cell cultures (12, 21, 22). Before cell seeding, scaffolds were protein-adsorbed by immersion in neural expansion medium for 12 hours in a 37˚C incubator. Cells at passages 10-20 were trypsinized, stained with trypan blue, counted, and then plated at a density of 3×105 cells/cm2 onto the pre-wetted matrices that had been placed into 24- well culture plates for at least 15 minutes to allow the cells to bind to the scaffold prior to adding neural expansion medium. After seeding, plates were incubated at 37˚C in a humidified 5% (v/v) CO_2_ atmosphere and the medium was changed every second day.

For SEM analysis, scaffolds were fixed in 2.5% GA in 0.1 M PBS (pH=7.4) overnight at 4˚C and post-fixed by 1% osmium tetroxide for 1.5 hours. Fixed samples were dehydrated through exposure to an ethanol gradient and allowed to air dry in a hood.

### Immunofluorescence staining

Cells were fixed overnight in 4% paraformaldehyde in 0.1 M PBS and processed for immunocytofluorescence staining. The washing step was performed 3 times, for 5 minutes each with 0.05% PBS-tween, then samples were permeabilized with 0.2% triton X-100 for 15 minutes and non-specific antigens were blocked with 10% normal goat serum (12036, SAFC Biosciences, USA) for 1 hour. Cells were then incubated overnight using the following primary antibodies: monoclonal mouse IgG anti-Nestin (1:100, MAB5326, Chemi-Con), monoclonal mouse IgG anti-Tubulin III/Tuj1 (1:500, T4026, Sigma-Aldrich), monoclonal mouse IgG anti-microtubule associated protein (MAP2, 1:200, M-1406, Sigma-Aldrich), polyclonal rabbit IgG anti-glial fibrillary acidic protein (GFAP, 1:250, Santa Cruz, SC-6171), or polyclonal rabbit IgG anti-SOX1 (1:300, 22572, Abcam, Cambridge, UK) for 1 hour at 37˚C. After the washing stages, cells were incubated with the following secondary antibodies: FITC anti-mouse IgG (1:400, AP308F, Chemi-Con) and FITC anti-rabbit IgG (1:400, F1262, Sigma-Aldrich) for 1 hour at 37˚C. Counterstaining was conducted using the nuclear dye propidium iodide (PI, P4864, Sigma-Aldrich) and 4, 6-diamidino-2-phenylindole (DAPI, 1 μg/ml, D8417, Sigma-Aldrich) for 3 minutes at room temperature. Labeled cells were examined with a fluorescent microscope (BX51, Olympus, Japan) and images were taken with a camera (Digital Camera System, DP70, Olympus, Japan).

To evaluate hiPSC-NPs in scaffolds, cells were fixed as above and processed for immunohistofluorescence staining by standard protocols for paraffin embedding and sectioned into 10 μm sections. The sections were processed for antigen retrieval. In antigen retrieval, the deparaffinized and rehydrated sections were subjected to 0.05% trypsin in distilled water and 0.1% calcium chloride. After this step, they were washed and processed for immunostaining as described above.

### Cell proliferation

Cells that proliferated on the collagen sponges were quantified using 3-(4, 5-dimethyl-2-thiazolyl)-2, 5-diphenyl-2H-tetrazolium bromide (MTT, M5655, Sigma-Aldrich). MTT was reduced by mitochondrial dehydrogenase to a purple formazan precipitate. Cellseeded scaffolds were briefly cultivated for periods of 1, 4, 7, and 14 days. At these specific time points, the culture medium was replaced by a solution of serumfree culture medium to MTT solution (5 mg/ml in PBS) at a ratio of 5:1. After incubation for 2 hours at 37˚C and 5% CO_2_, the purple colored formazan was dissolved with dimethyl sulfoxide (DMSO, D2650, Sigma-Aldrich) and absorbance was measured at 540 nm with an absorbance microplate reader (ELISA, Elx800, BioTek, USA). The unseeded collagen scaffold was used to rule out any background absorbance.

### Cell infiltration

For the analysis of cell migration within the scaffolds, NPs were carefully seeded on the surface of the scaffolds without using any external mechanical force in order to induce cell infiltration. After one week these samples were fixed in 4% paraformaldehyde, embedded in paraffin, and thin-sectioned with a microtome at a thickness of 10 μm. Random longitudinal sections from the samples were mounted onto glass slides and evaluated for the presence of cell infiltration. After deparaffinization the cell nuclei were stained with PI for 3 minutes at room temperature. Labeled cells were then examined with a fluorescent microscope and acquired images analyzed with Image J software.

### Statistical analysis

All results were expressed as mean ± standard deviation (SD). Comparisons between groups for collagen concentrations and freezing temperatures were performed by analysis of variance (ANOVA) and Tukey’s test, using SPSS software 16.0. Statistical significance was set at p<0.05.

## Results

### Characterization of collagen scaffolds

**Fig 1:**
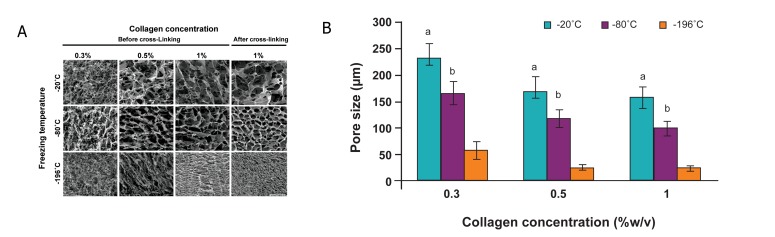
Characterization of collagen scaffolds at different fabrication parameters. A. SEM images of cross-section of uncrosslinked and cross-linked collagen scaffolds at different fabrication parameters. All images are at identical magnification. Scale bar: 200 μm. B. The effect of freezing temperatures on scaffold pore size in various collagen concentrations prior to cross-linking. a; p<0.001 -20˚C group vs. other groups. b; p<0.001 -80˚C group vs. -196˚C group.

Figure 1A shows the cross-sectional morphology of porous scaffolds by SEM that were fabricated using the freeze-drying process at various temperatures (20-80, and-196˚C) for different concentrations of collagen (0.3, 0.5, and 1% w/v). The structure of the freeze-dried scaffolds was foam-like and very porous. The pore wall became thinner with reducing collagen concentrations. We observed an interconnected network pore configuration and a high porosity throughout the cross-sections of all scaffolds. Quantification of pore size in all concentrations showed that lower temperatures led to smaller pore sizes (Fig 1B). The porosity of the scaffolds was >98%, which did not significantly change at various freezing temperatures (Table 1).

Table 1 and figure 1A show the properties of freezed-dried 1% w/v collagen scaffold before and after crosslink with GA. The pore size reduced after cross-linking from 98.5 ± 14.5 μm to 81.3 ± 18.6 μm. The porosity of the scaffolds was also >99% and did not change significantly after cross-linking. The cross-linking led to an increase in the swelling ratio. During cross-linking, GA reacted with free amine groups (Lys, Hylys) in collagen and caused a decrease in the free amine groups. Cross-linking caused approximately 21% shrinkage of the original volume and 93% mass retention.

**Table 1:** Characteristics of freeze-dried collagen sponges fabricated with 1% w/v and at varying freezing temperatures before and after cross-linking.


Sample	Temperature (°C)	Porosity (%)	Swelling ratio(%)	Number of free amine groups

**Before cross-linking**	-80	99.2 ± 0.1	94.9 ± 0.4	32.9 ± 1.7
**After cross-linking**	-20	99.1 ± 0.1	98.3 ± 0.4	-
	-80	99.1 ± 0.1	98.6 ± 0.3	12.9 ± 1.6
	-196	98.9 ± 0.1	98.6 ± 0.2	-


Table 2 shows the mechanical properties of collagen scaffolds with different collagen concentrations and cross-linking. All samples exhibited ductile and sponge-like behavior. Young’s modulus of scaffolds prepared from 0.3% collagen solution was only 0.2 MPa. This improved to 0.3 MPa at 0.5% collagen concentration and 0.7 MPa at 1% collagen concentration. The GA cross-linked scaffolds obtained from 1% collagen solution had a greater modulus (1.8 MPa) than the uncross-linked sample. The maximum load of the collagen sponges increased and the distensibility (strain at yield) reduced with increasing collagen concentration. However, they did not significantly change with cross-linking. Therefore, both the polymer concentration and cross-linking affected mechanical properties. According to our experimental design, any significant difference in the mechanical properties of scaffolds fabricated at the different freezing temperatures was not detected (data not shown).

**Table 2:** Mechanical properties of collagen scaffolds fabricated with 0.3, 0.5, and 1% w/v collagen concentration at -80°C in addition to the effect of GA cross-linking.


Samples	Modulus (MPa)	Maximum load (cN)	Tensile strain (%)

**0.3%**	0.2 ± 0.03	6.8 ± 0.5	23.1 ± 0.9
**0.5%**	0.3 ± 0.02	11.9 ± 0.6	25.3 ± 0.8
**1%**	0.7 ± 0.3	23.3 ± 1.6	11.0 ± 3.3
**1%, GA**	1.8 ± 0.1	24.2 ± 2.5	11.2 ± 2.3


### Cytocompatibility, proliferation, and infiltration of cells

**Fig 2:**
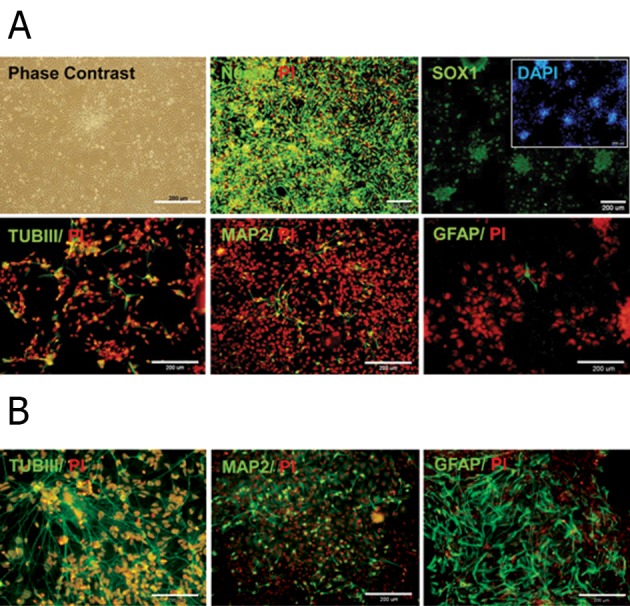
Characterizations of hESC-NPs. A. Representative phase contrast photomicrograph and immunofluorescence staining for NP markers, nestin and SOX1. At this step few cells expressed the neuronal markers, TUBIII and MAP2, and the astroglial marker, GFAP. B. We assessed the hESC-NPs potency to generate neuronal and glial derivatives by removing growth factors from the culture medium. This gave rise to spontaneous differentiation after 30 days. Immunofluorescence staining for neuronal markers, TUBIII and MAP2, and astroglial marker, GFAP.

In order to evaluate the scaffolds for neural tissue engineering we established a homogenous, expandable, and self-renewable population of multipotent NPs. These NPs were generated from hiPSCs by using an adherent system and a defined medium supplemented with a combination of growth factors (21). The adherent hiPSC-NPs culture stained uniformly for Nestin and SOX1, whereas a smaller number expressed mature neuronal markers (TUBIII and MAP2) and the astrocyte marker (GFAP, Fig 2A). The generated NPs have the potential to differentiate spontaneously by growth factor withdrawal and to produce neurons and astrocytes (Fig 2B). These data showed that our cells were homogenous and had the potential to differentiate into neuronal and glial cells.

**Fig 3:**
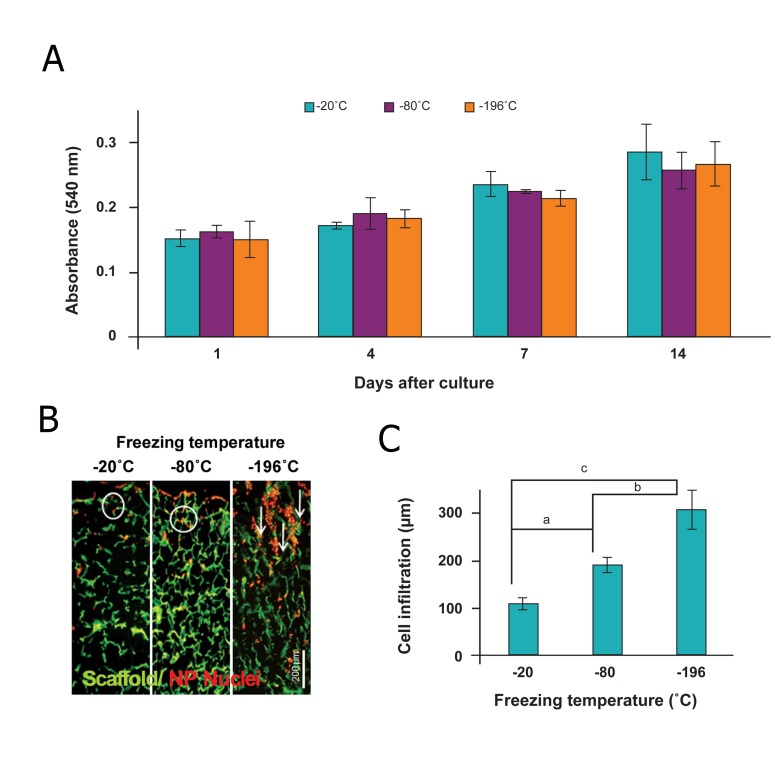
Behavior of hiPSC-NPs seeded on collagen scaffolds fabricated at various freezing temperatures and subsequently different pore structures. A. MTT assay after 1, 4, 7, and 14 days of cell seeding on collagen scaffolds prepared at temperatures of -20, -80, and -196˚C. Freezing temperature causes changes in pore structure that can affect cell proliferation as a biophysical cue of tissue-engineered scaffolds. B. Fluorescent staining of cell-seeded nuclei with PI. The observed green color is for scaffold autofluorescence. Cells oriented according to the pore orientations which are aligned for the scaffolds fabricated at -196˚C because of rapid freezing and water crystal orientation. C. Quantification of fluorescence pictures for comparing NPs infiltration into the scaffolds at various pore structures. a; p<0.05, b; p<0.01 and c; p<0.001.

hiPSC-NPs cultured on the collagen scaffold were assessed by MTT assay at days 1, 4, 7, and 14 and showed increased proliferation over time. Additionally, cells that proliferated on different collagen scaffolds that had been prepared under different freeze-dried conditions showed similar trends (Fig 3A).

Cells grew not only on the surface of the scaffolds, but also inside the freeze-dried porous collagen matrices as observed by multiple focal planes throughout the scaffold. As shown in figure 3B, seeded NPs oriented through the pore walls by "contact guidance" and aligned through the scaffolds fabricated at -196˚C which had oriented pores. Remarkably, NPs migrated about 309 ± 41 μm on scaffolds that were fabricated at -196˚C, which was approximately a three-fold greater distance compared to scaffolds fabricated at -20˚C which had circular pores (113 ± 13 μm, Fig 3C).

**Fig 4:**
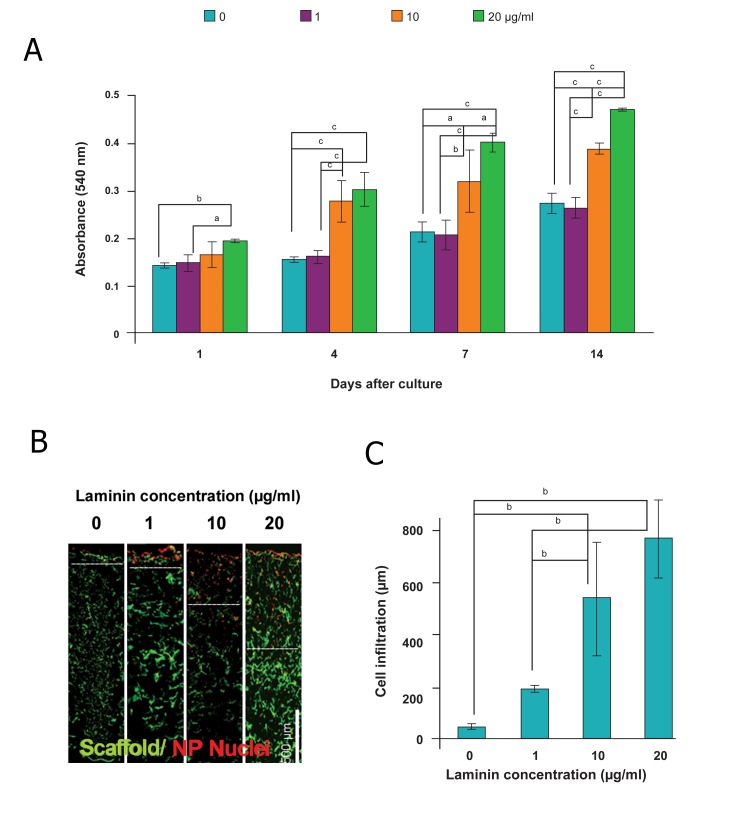
Behavior of hiPSC-NPs seeded on collagen scaffolds fabricated at -80˚C and coated with different laminin concentrations. A. MTT assay after 1, 4, 7, and 14 days after cell seeding on collagen scaffolds coated with 0, 1, 10, and 20 μg/ml laminin. Cell proliferation improved in a laminin concentration-dependant manner. B. Fluorescent staining of cell-seeded nuclei with PI. The scaffold autofluorescence are observed in green color. NPs migrated more into the scaffolds in the presence of laminin in a dose-dependent manner. C. The quantification of NPs that infiltrated into the collagen scaffolds. a; p<0.05, b; p<0.01 and c; p<0.001.

To evaluate the effect of laminin coating on the proliferation and migration of hiPSC-NPs, collagen scaffolds prepared at -80˚C were coated with different laminin concentrations (Fig 4). MTT results demonstrated greater absorbance at higher concentrations of laminin, which showed more cell attachment and proliferation on the evaluated days. The proliferation rate of NPs on 10 μg/ml laminin-coated scaffolds was 133% from days 1 to 14 and increased to 142% on 20 μg/ml laminincoated collagen scaffolds during the same time period which showed the higher proliferative capacity at higher concentrations of laminin (Fig 4A).

**Fig 5:**
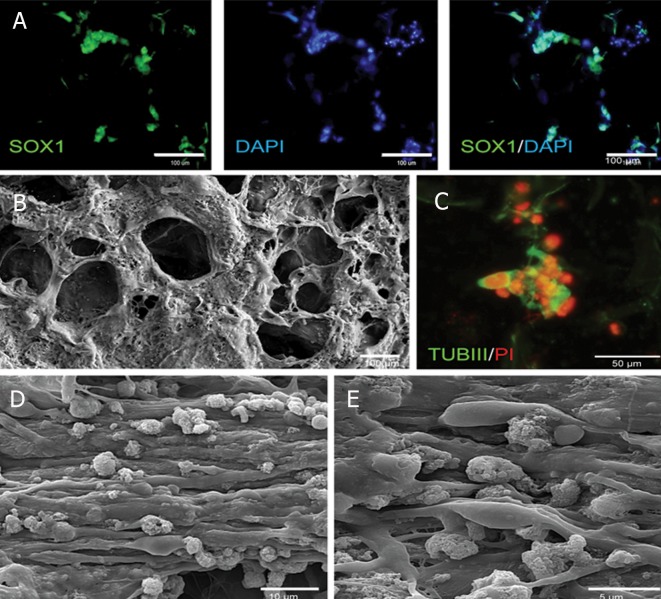
Immunofluorescence and scanning electron micrograph of cell-seeded scaffolds before and after differentiation. Cells were cultured on 1% collagen concentration, at -80˚C freezing temperature, and with GA cross-linked threedimensional collagen scaffolds. A. Immunofluorescence staining for SOX1, an undifferentiated NP marker one week after culture in an undifferentiated state. Nuclei were stained by DAPI (blue color). B. SEM one week after culture in an undifferentiated state. C. Immunofluorescence staining for TUJ1 after spontaneous differentiation of hiPSCNPs after four weeks. Nuclei were stained by PI (red color). D and E. SEM four weeks after spontaneous differentiation of hiPSC-NPs. Note the parallel arrangement of differentiated neuronal cells.

In the presence of 20 μg/ml laminin, cell infiltration inside the collagen scaffolds was promoted to 773 ± 149 μm, which was about 16 times higher than the cell infiltration into the uncoated collagen scaffolds (Fig 4B, C). Figure 5 shows immunofluorescence and SEM images of cell-seeded scaffolds before and after differentiation. We evaluated attachment, stemness, and differentiation of hiPSC-NPs on the surfaces of the collagen scaffolds (80˚C-1%) after cell seeding for one week of expansion and four weeks of differentiation. At this time, the NPs maintained the stemness marker, SOX1 (Fig 5A) and expanded on the surface of the scaffold (Fig 5B). Additionally, after growth factor withdrawal, NPs spontaneously differentiated into neuronal cells and expressed TUJ1 (Fig 5C). The neuronal processes were arranged in parallel (Fig 5D, E).

## Discussion

According to numerous studies cell-seeded collagen scaffolds are beneficial for the repair of injuries to the central (5) and peripheral nervous systems (23). The biodegradable, naturally-derived collagen scaffolds not only work as vehicles for cell transplantation, particularly for neuronal cells, but also their physical and biochemical signals may regulate adhesion, proliferation, differentiation, and function by seeded cells. For this reason, we have constructed collagen scaffolds at various freezing temperatures and used them to evaluate the proliferation and migration of hiPSC-NPs in three-dimensional scaffolds with different pore structures. Our data showed a reduction in pore size of the scaffolds at lower temperatures which related to more rapid ice formation after freezedrying (24). However, there was no significant change in porosity, swelling ratio and mechanical properties at the different freezing temperatures.

Since we wanted to assess this scaffold for neural tissue engineering, we used hiPSC-NPs to evaluate the cytocompatibility, proliferation, infiltration, and differentiation of cells in the collagen scaffolds. The cellular proliferation on these scaffolds with varying pore sizes and unchanged porosity, swelling ratio, and mechanical properties showed a similar trend.

With regards to the physical effects of these scaffolds, we observed that hiPSC-NPs showed more infiltration into the collagen scaffolds that have been prepared at lower freezing temperatures. Cell infiltration into scaffolds is a critical parameter affected by biochemical, mechanical and geometrical properties of a scaffold. This effect possibly was the result of the increased surface area or alignment of pores in those scaffolds fabricated at -196˚C, which showed the highest cell infiltration. Importantly, the alignment of NPs through oriented pores by contact guidance is highly desired for neural tissue engineering (11). The effects of freezing rate on the physical properties of collagen-glycosaminoglycan scaffolds and cell behavior have previously been studied (10, 25).

Coating of collagen scaffolds with laminin significantly increased proliferation and infiltration of the cells in a dose-dependent manner. This was consistent with the findings of other researchers who have shown that laminin when added to the medium (21) or coated on tissue culture plates (12) caused a reduction in apoptosis and enhanced NPs survival and proliferation. NPs migration into the scaffolds correlated with the concentration of laminin used as a coating, which was in agreement with other research groups who have reported that laminin enhanced NPs migration when coated on cover slips (22).

It seems that seeded cells translate scaffold signals into cell behavior changes through integrin (26). Integrins are cell surface receptors that mediate cell-ECM adhesion and signaling (27). Activation of integrins leads to the rearrangement of cytoskeletal components and the recruitment of kinases, such as focal adhesion kinase (FAK), and finally to activate the mitogen-activated protein kinase (MAPK) signaling pathway to promote gene transcription (28).

## Conclusion

Three-dimensional biodegradable scaffolds seeded with stem/progenitor cells provides one of the most interesting strategies in the field of biomaterials (29). This study has described the fabrication and cytocompatibility of porous collagen scaffolds by freeze-drying, and the effect of pore structure and laminin as biophysical and biochemical cues on hiPSC-NPs behavior. The laminincoated collagen scaffolds are good potential candidates for neural tissue engineering.
